# Malnutrition alters protein expression of KNDy neuropeptides in the arcuate nucleus of mature ewes

**DOI:** 10.3389/fphys.2024.1372944

**Published:** 2024-06-07

**Authors:** Jennifer F. Thorson, Ligia D. Prezotto

**Affiliations:** ^1^ Nutrition, Growth and Physiology Research Unit, U.S. Meat Animal Research Center, Agricultural Research Service, United States Department of Agriculture, Clay Center, United States; ^2^ Physiology Laboratory, Department of Animal Science, University of Nebraska-Lincoln, Lincoln, United States

**Keywords:** dynorphin A, ewe, hypothalamus, kisspeptin, KNDy, neurokinin B, nutrition, nutrient-sensing

## Abstract

The neuropeptides kisspeptin, neurokinin B, and dynorphin A are imperative for the pulsatile secretion of gonadotropin-releasing hormone and luteinizing hormone to ultimately regulate reproductive cyclicity. A population of neurons co-expressing these neuropeptides, KNDy neurons, within the arcuate nucleus of the hypothalamus (ARC) are positioned to integrate energy status from afferent neuronal and glial cells. We hypothesized that KNDy-expressing neurons in the ARC of mature ewes are influenced by energy balance. To test this hypothesis, ovary-intact, mature ewes were fed to lose, maintain, or gain body weight and hypothalamic tissue harvested during the luteal phase of the estrous cycle. Fluorescent, multiplex immunohistochemistry with direct antibody conjugation was employed to identify and quantify neurons expressing a single neuropeptide, as well as for the first time report co-expression of kisspeptin, neurokinin B, and dynorphin A protein in the ARC. Previous reports using this population of ewes demonstrated that concentrations of insulin and leptin differed between ewes fed to achieve different body weights and that ewes fed to gain body weight had increased concentrations of progesterone. Moreover, within this population of ewes tanycyte density and cellular penetration into the ARC was increased in ewes fed to gain body weight. Within the current report we have revealed that the number of neurons in the ARC expressing kisspeptin, neurokinin B, and dynorphin A protein was increased in ewes fed to gain body weight. Moreover, the number of KNDy neurons in the ARC expressing all three neuropeptides within a single neuron was decreased in ewes fed to lose body weight and increased in ewes fed to gain body weight when compared to ewes fed to maintain body weight. The cumulative findings of this experimental model suggest that expression of kisspeptin, neurokinin B, and dynorphin A protein in the ARC during the luteal phase of the estrous cycle are influenced by energy balance-induced alterations in circulating concentrations of progesterone that drive changes in morphology and density of tanycytes to ultimately regulate central perception of global energy status. Moreover, these results demonstrate that changes in KNDy neurons within the ARC occur as an adaptation to energy balance, potentially regulated divergently by metabolic milieu via proopiomelanocortin afferents.

## 1 Introduction

At the central level, the hypothalamic region of the brain is responsible for the synthesis and secretion of several neuropeptides that are involved in the control of energy homeostasis and reproductive function (Morton and Schwartz, 2001; Morton et al., 2005; Alves et al., 2015; Cardoso et al., 2015). The hypothalamus is essential in the integration of central and peripheral signals that are fundamental for the regulation of reproductive function. The axis of communication between the peripheral circulation and the brain is critical in the regulation of energy metabolism (Oomura et al., 1964). The lateral, paraventricular, dorsomedial, ventromedial, and arcuate (**ARC**) nuclei of the hypothalamus play specific and key roles in this regulation (Oomura et al., 1964; Morton et al., 2005). Within these hypothalamic nuclei, subsets of neurons with specific neurobiological phenotypes are responsive to glucose, fatty acids, amino acids, and other fuel-related stimuli (Kalsbeek et al., 2010). In these nutrient-sensing neurons, nutrients act as signaling molecules to engage a complex set of neurochemical and neurophysiological responses, thereby perceiving global energy status of the animal and thus determining if reproduction is a viable process in the current energy state.

The neuropeptide kisspeptin is a key regulator in reproductive function (Pinilla et al., 2012). Neurons expressing kisspeptin are present in two major neuronal populations, the preoptic region and the ARC. Interestingly, it has been demonstrated that most cells expressing kisspeptin are located within the ARC and also co-express the neuropeptides neurokinin B and dynorphin A (Goodman et al., 2007), landing this population of neurons the moniker **KNDy** neurons (Cheng et al., 2010). Both neurokinin B and dynorphin A within the ARC play critical roles in steroid feedback to gonadotropin-releasing hormone (**GnRH**) -expressing neurons and are thus integral to reproductive function (Rance and Bruce, 1994; Foradori et al., 2005). In fact, mutations to kisspeptin, neurokinin B, or their cognate receptors results in failure to attain puberty, impaired gonadotropin secretion, absence of secondary sex characteristics, and infertility (de Roux et al., 2003; Seminara et al., 2003; Topaloglu et al., 2009; Mei et al., 2011). The independent roles of kisspeptin, neurokinin B, and dynorphin A have been extensively examined in sheep in regard to regulation of the reproductive axis (Foradori et al., 2002; Smith et al., 2006; Porter et al., 2014; Merkley et al., 2020; Harlow et al., 2022), but no reports have investigated the influence of malnutrition on protein expression in neurons that express all three neuropeptides.

The role of KNDy neurons in nutrient-sensing seems plausible due to the anatomical structure and prime positioning proximal to the peripheral circulation and cerebral spinal fluid to gauge global metabolic status. Further, it is well documented that reproductive function is highly dependent upon metabolic status regulated by such factors as: free fatty acids, glucose, insulin-like growth factor I, and leptin (Goubillon and Thalabard, 1996; Conway and Jacobs, 1997; DiVall et al., 2010; Jungheim et al., 2011). Neurons expressing kisspeptin, neurokinin B, and dynorphin A express metabolic receptors (Backholer et al., 2010; Cernea et al., 2016), providing the necessary machinery for KNDy cells to mediate both negative and positive feedback effects of global energy status. Moreover, afferents of KNDy neurons (such as proopiomelanocortin (**POMC**) expressing neurons) also express metabolic receptors, including the leptin and insulin receptors (Elias et al., 1999; Cernea et al., 2016). Cumulatively, these data provide evidence that KNDy neurons and afferents may mediate the effects of nutritional status on the reproductive neuroendocrine axis—potentially through leptin-dependent POMC-mediated mechanisms. However, it remains to be determined how this mediation is regulated so we set out to test the hypothesis that altering plane of nutrition results in altered protein expression of kisspeptin, neurokinin B, dynorphin A, and their co-expression within the ARC of ovary-intact, mature ewes during the luteal phase of the reproductive cycle.

## 2 Materials and Methods

Animal care and use protocols were approved by the North Dakota State University Institutional Animal Care and Use Committee.

### 2.1 Animals and experimental design

Animal care and dietary treatments were as previously described (Kaminski et al., 2015; Bass et al., 2017). Briefly, a total of 48 Rambouillet-cross multiparous ewes (3–5 years of age and of similar genetic background) were housed individually (0.91 × 1.2 m pens) in a temperature-controlled room (14°C) with regulated light-dark cycle (12:12 h; lights on 0700 and off at 1900; initiated prior to the winter solstice). Animals were stratified by weight and then randomly assigned to one of three treatments: 200% of dietary recommendations (**overfed**; *n* = 16; based on NRC, 2007), 100% of dietary recommendations (**control**; *n* = 16), or 60% of dietary recommendations (**underfed**; *n* = 16), as previously described by Grazul-Bilska et al. (Grazul-Bilska et al., 2012). Diets were initiated 60 days before estrus synchronization (d −60). Animals were fed twice a day and weighed weekly to ensure target BW was achieved. By d 0, animals had achieved the desired BW that was maintained until the completion of the experiment (d 15). Animals were maintained in the treatment groups for a total of 75 days. Estrus was synchronized and blood samples collected on day 12 of the estrous cycle at 0700 following overnight feed withdrawal for harvest of serum for analysis of hormones and metabolites as previously described (Grazul-Bilska et al., 2012; Kaminski et al., 2015). Animals were euthanized via captive bolt and exsanguinated during the late-luteal phase of the estrous cycle. The presence of corpora lutea was visually confirmed in all ewes following euthanasia. For this experiment, a tissue block containing the hypothalamus was collected by making the following cuts: rostral to the optic chiasm, caudal to the mammillary body, lateral to the hypothalamic sulci, and dorsal to the anterior commissure. The tissue block was rapidly frozen in liquid nitrogen vapor and stored at −80°C until analysis.

### 2.2 Hypothalamus processing and immunohistochemistry

Coronal sections (20 μm) were cut from hypothalamic blocks from a randomly selected subset of the ewes (*n* = 7; two or three per treatment) using a cryostat, mounted on chrome-alum-gelatin-coated slides, and stored at −80°C until processed. All ewes in the subset consumed the entire ration offered for their respective treatment. A series of comparable sections within the ARC were used for detection of kisspeptin, neurokinin B, dynorphin A, and colocalization of the aforementioned neuropeptides within the same cell by immunohistochemistry as previously described (Mullier et al., 2010; Prezotto et al., 2020). Briefly, slide-mounted sections were thawed and air-dried at room temperature. Slides were fixed by immersion for 1 min at −20°C in methanol/acetone (1:1). Slides were washed twice in 0.1 M phosphate-buffered saline (PBS; pH 7.4) and then incubated overnight at 4°C with 0.3% Triton X-100 with rabbit anti-kisspeptin (1:50,000; gift from Alan Caraty; lot 564) conjugated to DyLight 488, rabbit anti-neurokinin B (1:500; Biorbyt; Cambridge, United Kingdom) conjugated to DyLight 550, and rabbit anti-dynorphin A (1:200; Phoenix Pharmaceuticals; Burlingame, CA) conjugated to DyLight 633. All antibody conjugation to fluorophores was performed using DyLight Conjugation Kits (AbCam; Boston, MA) according to manufactures recommendations, following the purification of antibodies with Serum Antibody Purification kits (AbCam; Boston, MA) according to manufacturer’s recommendations. Sections were then washed twice in 0.1 M PBS and incubated for 1 min with Hoechst (1:1,000; Thermo Fisher Scientific; Waltham, MA) diluted in 0.1 M PBS to counterstain cellular nuclei. Finally, slides were washed twice in 0.1 M PBS and coverslipped with a Mowiol mounting media containing 2.5% DABCO.

### 2.3 Imaging and analyses

Tissue sections were observed using ×10 and ×20 objectives with an epifluorescence microscope (Leica DM6 B; Leica Microsystems Ltd.) equipped with a motorized stage and a digital monochromatic camera (Leica DFC3000 G; Leica Microsystems Ltd.). Images were analyzed using the Leica Application Suite X (LAS X; Leica Microsystems Ltd.) software. Threshold and background signal was established and used to normalize all images within anatomical division of interest prior to analysis.

Localization of neuronal soma expressing protein integral to regulation of GnRH synthesis and secretion was performed using kisspeptin, neurokinin B, and dynorphin A-specific staining within the ARC of ewes. Colocalization of kisspeptin, neurokinin B, and dynorphin A was assessed to quantify the specific presence of the aforementioned neuropeptides within a single neuronal soma in the ARC. Within each section, a single ROI (400 µm diameter circle) was placed over the ARC adjacent to the third ventricle to quantify the number of each kisspeptin, neurokinin B, and dynorphin A-expressing neuronal soma (manually counted by a single, trained individual blinded to treatment) and the number of neuronal soma co-expressing kisspeptin, neurokinin B, and dynorphin A. Data collection was performed in comparable sections (9 sections/animal; 1 ROI/section) within the ARC of each animal.

### 2.4 Statistical analyses

The main effect of dietary treatment on cell counts were evaluated using the Proc GLM procedure of SAS (SAS Institute Inc.). The sources of variation were treatment, section, and their interaction. Least squares means was used to compare means if significant differences were detected. Data are presented as Least squares means plus or minus standard error. Significance was declared at *p* ≤ 0.05 and a tendency was reported if 0.05 < *p* ≤ 0.10.

## 3 Results

### 3.1 Morphometrics, metabolites, and hormones of the model

Changes in gross and cellular morphometrics and serum concentration of metabolites and hormones in response to dietary treatments for this model have been reported previously (Kaminski et al., 2015; Bass et al., 2017; Prezotto et al., 2020). Briefly, dietary treatments induced weight gain (9.6 ± 0.7 kg) in overfed ewes and weight loss (−13.9 ± 0.1 kg) in underfed ewes (*p* ≤ 0.02). Concentration of glucose was greater (*p* < 0.01) in overfed ewes (4.26 ± 0.14 mmol/L) when compared to underfed ewes (3.75 ± 0.12 mmol/L), while control ewes (4.06 ± 0.09 mmol/L) were intermediate. Concentration of insulin was also affected by diet (*p* < 0.001) being greater in overfed ewes (112.7 ± 11.5 pmol/L) and reduced in underfed ewes (31.6 ± 2.9 pmol/L) when compared to control ewes (63.9 ± 5.7 pmol/L). Concentration of leptin was also affected by diet (*p* < 0.001) being greater in overfed ewes (12.6 ± 1.0 μg/L) and reduced in underfed ewes (3.0 ± 0.1 μg/L) when compared to control ewes (5.5 ± 0.3 μg/L). Moreover, number of corpora lutea as an indicator of ovulation rate was greater (*p* < 0.05) in overfed ewes (1.99 ± 0.12) and control ewes (1.70 ± 0.10) when compared to underfed ewes (1.24 ± 0.08), while only ewes fed to gain body weight had increased (*p* < 0.01) concentrations of progesterone (7.09 ± 1.08, 6.39 ± 1.11, and 10.34 ± 0.92 nmol/L for underfed, control, and overfed ewes, respectively). Finally, the number of POMC-expressing neurons within the ARC was increased (*p* ≤ 0.002) in underfed ewes (71.5 ± 3.3) when compared to control (49.5 ± 3.4) and overfed (56.2 ± 3.3) ewes, while the paraventricular tanycyte density and cellular penetration into the ARC (marker of hypothalamic barrier function and intercellular nutrient transport) was increased (*p* ≤ 0.002) in ewes fed to gain body weight (2,626,079 ± 127,071 pixels; 910 ± 23 µm) when compared to control (2,181,687 ± 127,071 pixels; 807 ± 23 µm) and underfed (2,110,977 ± 127,071 pixels; 769 ± 23 µm) ewes.

### 3.2 Kisspeptin, neurokinin B, and dynorphin A protein expression in neuronal soma within the ARC

**FIGURE 1:**
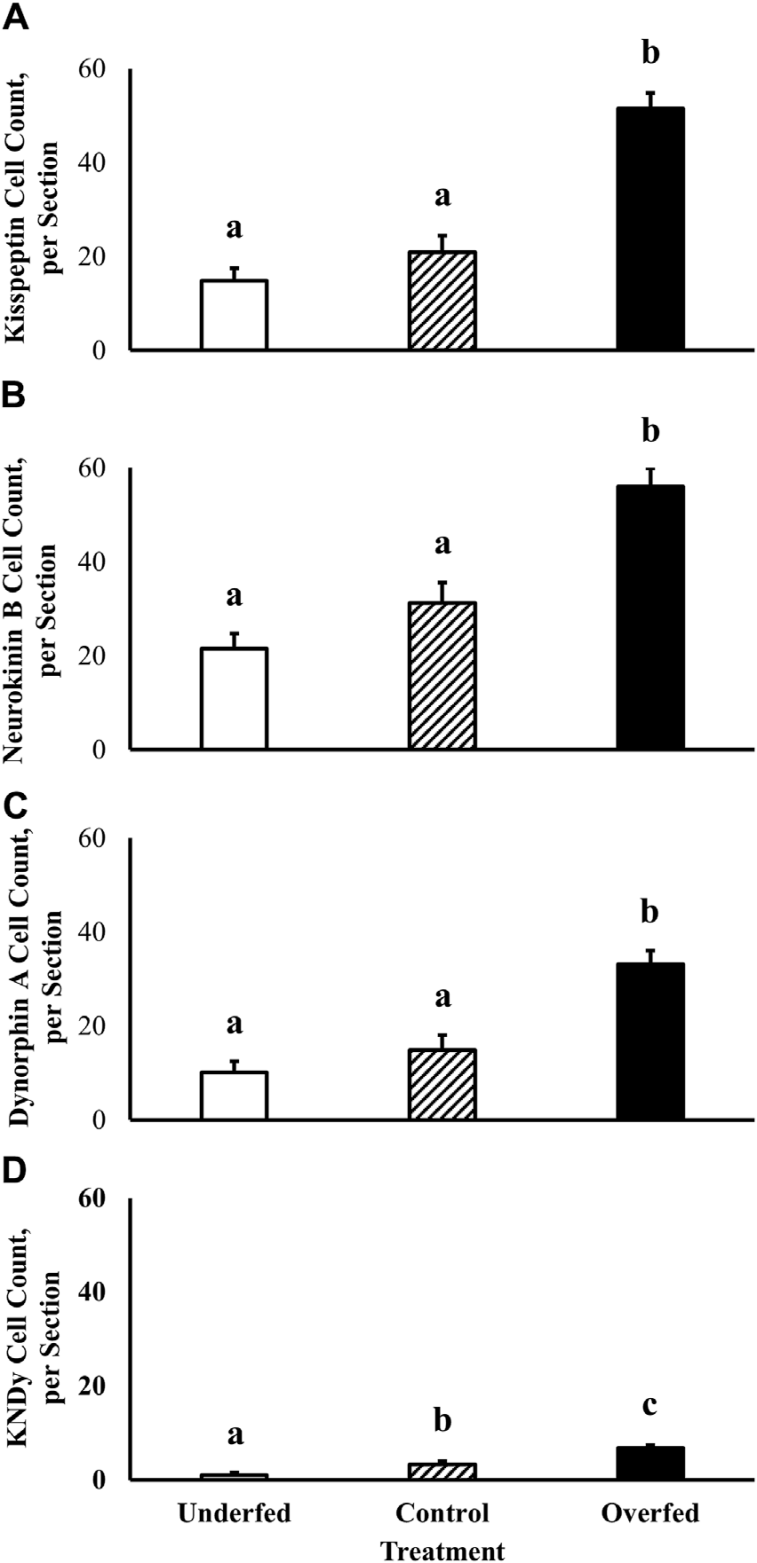
Kisspeptin, neurokinin B, and dynorphin A protein expression and co-expression (KNDy neurons) within the arcuate nucleus of the hypothalamus is under the influence of dietary treatment in mature ewes. The effect of plane of nutrition on the expression of neuropeptides that regulate GnRH synthesis and secretion [kisspeptin **(A)**, neurokinin B **(B)**, and dynorphin A **(C)**] and their colocalization **(D)** within the arcuate nucleus of the hypothalamus was tested in mature ewes. Dietary treatments were: 60% (underfed), 100% (control), and 200% (overfed) of dietary recommendations. Number of kisspeptin, neurokinin B, and dynorphin A-immunolabeled cells was greater (Treatment *p* < 0.0001) in overfed animals when compared with underfed and control groups. Number of KNDy-immunolabeled cells was reduced (*p* = 0.0092) in underfed ewes and increased (*p* = 0.0005) in overfed ewes when compared with the control group.

Localization of neuronal soma expressing proteins integral to regulation of GnRH synthesis and secretion was performed using kisspeptin, neurokinin B, and dynorphin A-specific staining within the ARC of ewes. Number of neurons in the ARC expressing kisspeptin (14.9 ± 2.7 neurons, 20.9 ± 3.6 neurons, and 51.5 ± 3.3 neurons in ewes fed to lose, maintain, and gain body weight, respectively), neurokinin B (21.5 ± 3.2 neurons, 31.3 ± 4.3 neurons, and 56.0 ± 3.9 neurons in ewes fed to lose, maintain, and gain body weight, respectively), and dynorphin A (10.1 ± 2.4 neurons, 14.9 ± 3.2 neurons, and 33.1 ± 2.9 neurons in ewes fed to lose, maintain, and gain body weight, respectively) protein was increased (*p* < 0.0001) in ewes fed to gain body weight (Figures 1A–C). However, there was no influence of section (*p* ≥ 0.21) or treatment by section interaction (*p* ≥ 0.23) on number of kisspeptin, neurokinin B, or dynorphin A-containing neurons.

### 3.3 KNDy protein expression in neuronal soma within the ARC

For the first time, we have demonstrated the co-expression of kisspeptin, neurokinin B, and dynorphin A protein within a single neuronal soma. As expected, kisspeptin, neurokinin B, and dynorphin A protein are localized to the cytoplasm of neuronal soma and absent within the nucleus. The colocalization of kisspeptin, neurokinin B, and dynorphin A (KNDy) was assessed to quantify the specific overlap of the aforementioned neuropeptides within a single neuronal soma in the ARC in order to assess the influence of dietary treatment. Number of KNDy neurons in the ARC expressing kisspeptin, neurokinin B, and dynorphin A protein was decreased in ewes fed to lose body weight (1.0 ± 0.5 neurons; *p* = 0.01) and increased in ewes fed to gain body weight (6.7 ± 0.6 neurons; *p* = 0.0005) when compared to ewes fed to maintain body weight (3.3 ± 0.7 neurons; Figure 1D). However, there was no influence of section (*p* = 0.48) or treatment by section interaction (*p* = 0.49) on number of KNDy neurons.

## 4 Discussion

The use of animal models has been critical in examining regulation of feed intake, energy balance, and functionality of the reproductive axis in response to energy intake (Allen et al., 2005; Clarke et al., 2008; Kanasaki and Koya, 2011; Caron et al., 2012). The neuropeptide kisspeptin is a key regulator in reproductive function (Pinilla et al., 2012). Moreover, both neurokinin B and dynorphin A within the ARC play critical roles in steroid feedback control of GnRH secretion. Cumulatively, the KNDy neuronal population therefore possess the ability to serve as a central node capable of integrating reproductive and nutritional functions. The independent roles of kisspeptin, neurokinin B, and dynorphin A have been extensively examined in sheep in regards to regulation of the reproductive axis (Foradori et al., 2002; Smith et al., 2006; Porter et al., 2014). However, only limited work in rodents and a single report in immature, ovariectomized, underfed sheep has been conducted regarding nutritional regulation of reproductive function via KNDy neurons (Caron et al., 2012; Harlow et al., 2022). Harlow et al. (2022) have reported that undernutrition reduces kisspeptin mRNA in neurons expressing kisspeptin and neurokin B. It has been proposed that a high percentage of cells expressing only two of the three KNDy neuropeptides can be classified as KNDy cells (Goodman et al., 2013). However, the current report that assessed protein expression of kisspeptin, neurokinin B, and dynorphin A individually as well as the protein co-expression of kisspeptin, neurokinin B, and dynorphin A indicate that at least at the protein level true KNDy neurons only represent a small portion of cells (approximately 13%). This small proportion is considerably lower than estimated in prior reports; however, it must also be noted that the endocrine environments differ between the current and prior reports in sheep (Goodman et al., 2007; Cheng et al., 2010; Merkley et al., 2020; Harlow et al., 2022). Moreover, we acknowledge that the population size for the current dataset is limited; however, the design and methodology of the current report profoundly limited extraneous factors and thus allowed us to identify differences attributed to nutritional status on the KNDy neuronal population. Furthermore, the current results have a profound influence on the implications of prior experiments as well as the design of future experiments assessing KNDy neuronal physiology under different physiological conditions.

Behaviors that characterize feed intake and maintenance of energy balance are regulated via a homeostatic system that include central and peripheral organs. At the central level, the hypothalamic region of the brain is responsible for the synthesis and secretion of several neuropeptides [neuropeptide Y (**NPY**), agouti-related protein, POMC, and α-melanocyte stimulating hormone] that maintain the balance between feed intake, energy expenditure, and nutrient storage (Kalra et al., 1999; Schwartz et al., 2000; Hillebrand et al., 2002). Maternal nutrient restriction throughout fetal development has detrimental effects on offspring performance (McMillen et al., 2005; Levin, 2006; Plagemann, 2006). Further, alterations in maternal dietary protein and undernutrition alter synaptic density and function (Smith and Druse, 1982; Torrero et al., 2014). Therefore, exposure to nutritional stress *in utero* may modulate development of nutrient-sensing neurocircuitry and subsequent feed intake and energy balance in the offspring. Functional studies have demonstrated that infusion of kisspeptin reduces the expression of POMC, while increasing NPY gene expression illustrating a functional connection between the reproductive and satiety axes (Backholer et al., 2010). This finding is astonishing considering a recent report in ewes within the luteal phase of the estrous cycle that demonstrates only approximately 2% of POMC expressing cells express the kisspeptin receptor (Goodman et al., 2023). Exogenous administration of leptin stimulates the expression of SOCS-3 (an intracellular inhibitor of leptin signaling) in a majority of POMC cells (Elias et al., 1999). Neurons expressing kisspeptin receive afferent input from POMC cells and thus possess the necessary machinery for the metabolic environment to gate reproductive function. In the current model we have reported that undernutrition increases POMC protein expression. Similarly, others have also reported increased POMC gene expression when ewes are offered a restricted diet (Sebert et al., 2009).

Global nutrient restriction inhibits kisspeptin gene expression and concentrations of luteinizing hormone in rodents, while kisspeptin treatment alleviates undernutrition-induced delayed puberty in female rats (Castellano et al., 2005). Developmental overnutrition also increases deleterious effects on the reproductive axis, such as advanced pubertal onset, irregular estrous cycles, and reduced number of liters produced (Caron et al., 2012; Connor et al., 2012). Studies have investigated the expression and function of neurokinin B by itself or in combination with kisspeptin or dynorphyn A (Goubillon et al., 2000; Foradori et al., 2006). Most of these studies have focused on how neurokinin B plays a role in reproduction of different female species. However, some have demonstrated that neurokinin B might also play an important metabolic role in thermoregulation. When expressed with kisspeptin, in animals that can not regulate activation of brown adipose tissue in order to produce heat, a reduced amount of calories are burnt by the animal (Navarro, 2020). Intracerebroventricular infusion of dynorphin A induces feeding, but blocked the satiety-induced effects of rumen distension and increased intraruminal concentration of propionate (Baile and McLaughlin, 1987; Della-Fera et al., 1990). Therefore, dynorphin A neurons are capable of responding to changes in nutritional status and transferring that information to GnRH cells.

Direct GnRH neuronal glucose-sensing has been evaluated by challenging glucose-sensing pathways and observing the responses of GnRH neurons (Roland and Moenter, 2011). However, none of these glucose-sensing mechanisms have been demonstrated to be directly present on GnRH neuronal cells. The role of KNDy neurons in nutrient-sensing seems plausible due to the anatomical structure and prime positioning proximal to the peripheral circulation and cerebral spinal fluid to gauge global metabolic status. Further, it is well documented that reproductive function is highly dependent upon metabolic status regulated by such factors as insulin-like growth factor I, leptin, and free fatty acids (Conway and Jacobs, 1997; DiVall et al., 2010; Jungheim et al., 2011). Neurons expressing kisspeptin express metabolic and stress receptors (leptin, glucocorticoid, etc.; Takumi et al., 2012; Smith et al., 2006), providing the necessary machinery for KNDy cells to mediate both negative and positive feedback effects of global energy status. In fact, kisspeptin neurons make reciprocal connections with NPY(orexigenic) and POMC (anorexigenic) cells that meter feed intake and behavior (Backholer et al., 2010). Moreover, infusion of kisspeptin reduces the expression of POMC, while increasing NPY gene expression illustrating a functional connection between the reproductive and satiety axes (Backholer et al., 2010). Structurally, NPY fibers lie in close proximity to kisspeptin neurons in the prepubertal heifer, but proximal fiber density is not altered by nutritional regimen (Alves et al., 2015). Interestingly, in the prepubertal heifer model NPY fibers lie in close apposition to GnRH neurons and thus also have the potential to directly regulate the GnRH axis. In contrast to reports of POMC RNA expression, protein expression of POMC increases due to nutrient restriction as previously reported in this model and by others (Sébert et al., 2009). This dicotomy is likely attributed to reduced POMC-derived peptide sectretion in the face of reduced satiety in underfed ewes. Cumulatively, these data provide evidence that KNDy neurons may mediate the effects of nutritional status on the reproductive neuroendocrine axis in higher species. However, it remains to be determined how this mediation is regulated.

Molecular traffic between peripheral circulation and the central nervous system is regulated by the blood-brain barrier, which is composed of endothelial cells that form tight junctions lining microvessels (Neuwelt et al., 2011) and the ventricular system (Langlet et al., 2013). Interestingly, fasting-induced hypoglycemia modifies the morphology and permeability of the blood-brain barrier (Langlet et al., 2013) and thus the conductance of metabolic signals transmitted to the central nervous system. Moreover, reorganization of tight-junction proteins and consequently capillary fenestration alter activity of hypothalamic neurons (Thorens and Larsen, 2004; Levin et al., 2011). Therefore, intrauterine programming of metabolic state (insulin resistance, adiposity; Jaquet et al., 2000; Yan et al., 2010) may alter the morphology of the barrier surrounding the brain and thus central homeostatic regulation. Langlet et al. (2013) has demonstrated that fasting-induced reductions in circulating concentrations of glucose induce the reorganization of tight junction proteins between tanycytes and increase the permeability of microvasculature within the ARC and therefore alter the molecular permeability of the ARC. These modifications were rescued following glucose infusion, thus illustrating glucose-specific plasticity within the hypothalamus. As key neuropeptides regulating nutritional and reproductive function are located within the ARC (Alves et al., 2015; Cardoso et al., 2015), alterations in molecular permeability of the ARC may have significant impacts on energy metabolism and reproductive function. Additionally, Si et al. (2014) have reported that administration of progesterone reduces blood-brain barrier permeability in a rodent model and thus providing an additional means to regulate blood-brain barrier permeability in a malnourished model. Moreover, reproductive function and energy balance of the ewe is likely regulated in part through glucose sensing occurring within the brainstem. Previous reports have identified glucose-responsive neurons in the brainstem using rodent models (Adachi et al., 1995; Yettefti et al., 1997), but no data have been reported in an ovine model. A recent report by Nakamura et al. (2024) has however demonstrated a role of glucose sensing in the brainstem on the reproductive axis in a caprine model. For this body of work, we tested the hypothesis that altering plane of nutrition results in the structural reorganization of the blood-brain barrier (capillary fenestrations and tanycyte cellular morphology) within the medial basal hypothalamus of adult animals (Prezotto et al., 2020). Proopiomelanocortin cellular content within the ARC was also assessed to test whether reduced nutritional status improved access of nutrients into the ARC, while decreasing the access of nutrients of overfed animals. And finally, circulating concentrations of progesterone were determined to assess the influence of nutrition on reproductive endocrinology. The results of this research demonstrate that nutritional plane modifies structures of the blood-brain barrier - that meter the transport of nutrients to nutrient-sensing neuronal circuitry. Furthermore, these results indicate that changes within the satiety center and endocrine milieu occur as an adaptation to nutrient availability. These dietary-induced changes on blood-brain barrier permeability are potentially conveyed by alterations in concentration of progesterone to ultimately regulate the perception of metabolic state by POMC neurons.

The cumulative findings of this and prior experiments using a malnourished ewe model suggest that expression of kisspeptin, neurokinin B, and dynorphin A protein in the ARC during the luteal phase of the estrous cycle are influenced by energy balance-induced alterations in circulating concentrations of progesterone that drive changes in morphology and density of tanycytes. Moreover, these results demonstrate that changes in KNDy neurons within the ARC occur as an adaptation to energy balance, potentially regulated divergently by metabolic milieu.

## Data Availability

The raw data supporting the conclusion of this article will be made available by the authors, without undue reservation.
